# When Project Commitment Leads to Learning from Failure: The Roles of Perceived Shame and Personal Control

**DOI:** 10.3389/fpsyg.2018.00086

**Published:** 2018-02-06

**Authors:** Wenzhou Wang, Bin Wang, Ke Yang, Chong Yang, Wenlong Yuan, Shanghao Song

**Affiliations:** ^1^Department of Human Resource Management, Business School, Beijing Normal University, Beijing, China; ^2^Department of Business Administration, Asper School of Business, University of Manitoba, Winnipeg, MB, Canada

**Keywords:** learning from failure, project commitment, personal control, shame, proactive behavior

## Abstract

Facing a remarkably changing world, researchers have gradually shifted emphasis from successful experiences to failures. In the current study, we build a model to explore the relationship between project commitment and learning from failure, and test how emotion (i.e., perceived shame after failure) and cognition (i.e., attribution for failure) affect this process. After randomly selecting 400 firms from the list of high-tech firms reported by the Beijing Municipal Science and Technology Commission, we use a two-wave investigation of the employees, and the final sample consists of 140 teams from 58 companies in the technology industry in mainland China. The results provide evidence for the positive role of personal control attribution in the relationship between project commitment and learning from failure. However, in contrast to previous studies, perceived shame, as the negative emotion after failed events, could bring desirable outcomes during this process. Based on the results, we further expand a model to explain the behavioral responses after failure, and the implications of our findings for research and practice are discussed.

*The failures and reverses which await men* - *and one after another sadden the brow of youth - add a dignity to the prospect of human life, which no Arcadian success would do.*

—Henry David Thoreau

## Introduction

Failures are inevitable in a rapidly changing commercial environment (Allchin, 2012; Hong and Lin-Siegler, 2012; Lin-Siegler et al., 2016). Although failure is regarded as a special source for learning (Drucker, 1993), people usually hold a negative and evasive attitude toward experiencing failure, and even avoid mentioning past failures (Cope, 2011). Entrepreneurs and scholars are more prone to seek constructive information from successful cases rather than failed experiences (Ellis and Davidi, 2005). Recently, with the vanishing of a stable environment, researchers have started to shift emphasis from learning from successful experiences to failed experiences (Kapur, 2008; Potter, 2013; Bolinger and Brown, 2015; Simpson and Maltese, 2017). This can provide more valuable information and can prevent individuals or organizations from failing again for the same reason (Ford, 1999; Labib and Read, 2015). Following past research, learning from failure is defined as ‘the sense that one is acquiring, and can apply, knowledge and skills’ after failure (Shepherd et al., 2011).

During the past several decades, previous studies have demonstrated that learning from failure is related to desirable outcomes for organizations (e.g., better structure of knowledge: Madsen and Desai, 2010; lower risk of future failure: Kim and Miner, 2007) and individuals (e.g., better innovation performance: Nguyen and Saetre, 2015; better financial performance: Boss and Sims, 2008). However, Shepherd (2003) proposes that people may not always take proactive measures (e.g., learning) after failure, but become obsessed with failures or show avoidance behavior, calling for the need to exploring the antecedents of learning from failure. Past studies have found that personal traits and experiences exert a considerable influence on an individual’s behavior after failures (Boss and Sims, 2008; Bolinger and Brown, 2015). For instance, Boss and Sims (2008) find that self-leadership can help individuals move toward recovery. Shepherd et al. (2011) find that project failure and coping orientation are both positively related to learning from project failure. However, rare empirical studies pay attention to the emotional bond (i.e., commitment), the psychological link between employees and their goals (Mowday et al., 1979; Klein et al., 1999), projects, and organizations (Dvir et al., 2004). As for the current study, the emotional bond between employees and their projects—project commitment—refers to one’s belief in the project goals and values, and the desire to engage in the project and to be a member of it (Mowday et al., 1979). After project failure, which means failing to meet project goals, employees with a high level of project commitment will take a series of measures to achieve project goals in the future and avoid failing again, such as learning from failure. In fact, previous empirical studies demonstrated that individuals with high levels of commitment would show more proactive behavior (Feather and Rauter, 2004) and struggle for the success of their goals, projects, and organizations (Dvir et al., 2004; Hoegl et al., 2004). However, there is still no study exploring the relationship between project commitment and learning from failure. Thus, we propose that project commitment is positively related to learning from failure.

Besides, scholars have proposed that emotion and cognition may both play important roles in human behavior (Dolan, 2002; Phelps, 2006). Although various organizational theories pay attention to an individual’s cognitive dimensions, cognitive theories regard people as emotion-free actors during the learning process (Catino and Patriotta, 2013). Some scholars even believe that emotion is negatively related to rationality, thereby exerting detrimental effects on learning (Simpson and Marshall, 2010). Zhao and Olivera (2006) firstly posit a framework of error reporting (i.e., failed events) to describe individual responses after error, including detection, situation assessment, and behavioral response. During the detection phase, people usually analyze the causes of failures, and emotions arise during the situation assessment phase. However, past empirical research has usually examined the role of emotion and cognition separately in the process of learning from failure (Russell et al., 1987; Catino and Patriotta, 2013; Mantere et al., 2013; Jenkins et al., 2014), but few combine them in a single study.

**FIGURE 1:**
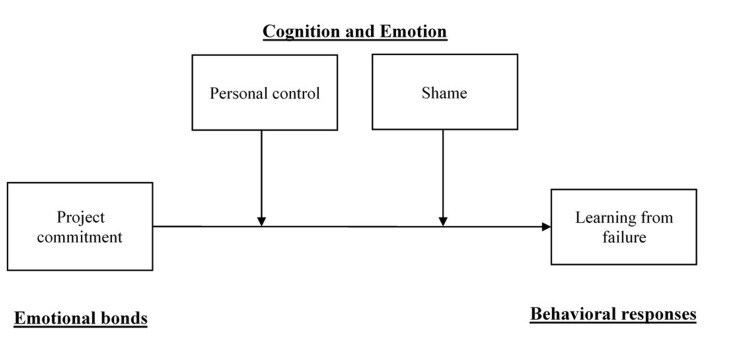
Theoretical model.

Consequently, in this paper, to further explain when people show a certain type of behavioral response—learning from failure, as shown in **Figure 1**—we developed a model, employing emotive and cognitive factors as boundary variables. From the resource perspectives (Quinn et al., 2012), we further developed Zhao and Olivera’s model to explain behavioral responses after failed events— emotional bond determines the total resource to use after failure, while emotion (i.e., shame) and cognition (i.e., personal control) have impacts on resource dividing tactics. Our results will shed light on the role of cognition and emotion in the relationship between project commitment and learning from failure, as well as providing various practical implications.

### Project Commitment and Learning from Failure

Project commitment was first defined as ‘the acceptance of and the strong belief in the goals and values of the project, the willingness to engage in the project, and the desire to maintain membership in the project’ (Mowday et al., 1979), which means team members with high commitment regard achieving project goals as their obligation (McDonough, 2000). For this reason, they will use their initiative to take extensive measures to pursue project goals, such as learning new skills, adjusting their behaviors, and so on (Hoegl et al., 2004).

Although success, like a beacon, provides individuals with potentially feasible solutions, failure could also become an important learning source (Shepherd et al., 2011, 2014). In fact, failure could provide valuable information (Corbett et al., 2007), including exposing their weaknesses in ability (e.g., time managing ability), knowledge (e.g., outdated industry knowledge), decisions (e.g., the allocation of resources), and so on. Besides, team members could also acquire new knowledge through reflecting on failure (McGrath, 2001; Shepherd et al., 2011). Due to such benefits, team members with high project commitment have stronger motivation to reflect past project failure, adjust their behavior to realize project goals, and achieve better performance. Therefore, they will make more efforts to learn from their failure.

Additionally, existing studies show that individuals with high levels of commitment are more willing to make extra contributions to the team, such as attending group activities, and sharing knowledge, because they usually hold the view that they must contribute information and knowledge to their teams (Jarvenpaa and Staples, 2001). Thus, they will actively participate in team activities of knowledge management and knowledge sharing (Hislop, 2003). Their learning and knowledge abilities, therefore, will be developed through these procedures, resulting in more efficient learning. Besides, by positively taking part in these activities, which focus on reflecting project experience, individuals not only share their own experience and knowledge, but also benefit from that of others, thereby learning more from past project failure (e.g., finding appropriate solutions to potential problems) through recombining multiple-angle thoughts (Jones et al., 2003; Yang, 2007; Terzieva and Morabito, 2016). Hence, we propose the following:

Hypothesis 1: Team members who have high project commitment learn more from failure than team members who have low project commitment.

### The Moderating Role of Shame

There is no denying that individuals will hold negative emotions such as guilt, anger, and shame when they fail (Carver and Scheier, 1990). Shame is one of the most important emotions (Zhao and Olivera, 2006; Bohns and Flynn, 2013), and it refers to a ‘self-conscious’ emotion, which will lead to more self-evaluation about the deviation between the current situation and the ideal situation (Tracy and Robins, 2006). Bohns and Flynn (2013) posit that shame may make people feel incompetent and inadequate. However, some scholars illustrate the difference in the cognition of shame between the East and the West (Bedford and Hwang, 2003). In fact, shame in Eastern (Confucian) cultures is associated with the relational self and morality, and it could be categorized as public shame and private shame, while shame is only associated with the individual self in Western Cultures (Bedford and Hwang, 2003).

Thus, individuals in Eastern cultures not only care about their self-evaluations but, also, other aspects which are strongly related to them. In other words, an individual’s glory and shame are associated with the teams they are working for. As stated above, individuals with high project commitment have strong emotional bonds related to the project’s team, and they always care about the team’s reputation. The emotion of shame after failure increases sensitivity toward others’ evaluations on the project team and makes the individual perceive more loss of reputation caused by the project failure. Therefore, they will take more effective actions to change others’ attitudes toward their teams (Elison and Partridge, 2012), such as reflecting, attending training programs, and so on, which could make them learn more from failure (Terzieva and Morabito, 2016).

In contrast, individuals with a low level of shame are less sensitive to negative evaluations of their teams. As a result, even if they have high project commitment, they have weaker motivation to make efforts to reflect on their past project experience and prevent failure for the same reason in the future. Therefore, the effectiveness of project commitment in generating learning from failure for individuals with a low level of shame will be weaker. Hence, we propose the following:

Hypothesis 2: Shame moderates the positive relationship between project commitment and learning from failure in such a way that the relationship will be stronger when shame is higher, rather than when it is lower.

### The Moderating Role of Personal Control

According to the attribution theory, individuals have different attribution patterns for achievement-related events (Standing et al., 2006; Mantere et al., 2013). For instance, Mantere et al. (2013) first employ narrative techniques to explore organizational stakeholders’ psychological process after venture failure, and the results show that attributions are associated with self-justification, which will influence emotional recovery after failure. Personal control attribution is regarded as one of the major attribution patterns which refers to the perceived controllability of cause (Judge and Bono, 2001; Shepherd, 2003; Shepherd et al., 2009). Following previous studies, personal controllability is one of the typical causal perceptions of the achievement-related events (Weiner, 1985), which is defined as an individual’s belief regarding the extent to which they can control causes (Russell, 1982; Russell et al., 1987). In others words, when facing deviation between performance and ideal performance, individuals with high perceived personal controllability believe that the cause of deviation is under their control and can be regulated (Banai et al., 2004). Past studies also demonstrate that personal control is an important antecedent of an individual’s subsequent psychological state, cognition, and behaviors (Anderson and Arnoult, 1985; McAuley et al., 1992).

Bandura and Wood (1989) first found that perceived controllability is positively related to self-efficacy (Judge and Bono, 2001). Therefore, team members who attribute failure to factors which are under their control are more likely to have a higher level of self-efficacy to overcome these causes of failure (Bandura and Wood, 1989; Martocchio and Dulebohn, 1994). In fact, past studies show that self-efficacy is positively associated with learning efficiency, thereby reinforcing the relationship between project commitment and learning from failure (Hearrington, 2010). Besides, as high perceived controllability would let individuals firmly believe that they have the competence to avoid failure (Judge and Bono, 2001), they will focus on themselves rather than seeking help from others or avoiding past failed events (Hutchinson et al., 2008). Zhao and Olivera (2006) also propose that, during the detection phase, the analysis of a failed project determines the directions or focus of subsequent behavior, while project commitment determines the level of effort that people will invest in. For instance, if people feel the causes of failure are out of their control, they may choose to seek help outside their team rather than learning from themselves. Therefore, they will spend more resources on themselves, including time, emotional resources, and cognitive resources.

In contrast, individuals with a lower level of perceived personal controllability usually hold negative attitudes toward the project failures. They believe that the effort for the goals is in vain, so that they have lower motivation to make more effort (Litt, 1988). Therefore, even if team members have a high level of project commitment, they may believe that failure is inevitable, rather than taking proactive behavior, such as learning and adjusting their behavior depending on the past project experience. Hence, we propose the following:

Hypothesis 3: Personal control moderates the positive relationship between project commitment and learning from failure in such a way that the relationship will be stronger when personal control is higher, rather than lower.

## Materials and Methods

### Participants and Procedure

We conducted a two-wave survey of high-technology firms in Beijing. We chose the high-tech industry because research and development (R&D) teams tend to have significant experience of failure in their work. To be accredited as a high-tech firm, it must have 60% of its annual sales from high-tech products (services) in the past year and at least 10% of its employees in R&D, among other criteria. Following the back-translation method (Brislin, 1980), all scales were first translated from English into Chinese and then back-translated into English by two independent individuals to ensure that no misinterpretations would arise from translation.

In 2016, we randomly selected 400 firms from the list of high-tech firms reported by the Beijing Municipal Science and Technology Commission. Our research assistants phoned each CEO (or Chairman) of the selected firms to elicit their firm’s participation. For the firms that agreed to participate, the CEOs and the research assistants jointly identified a coordinator; the research assistants and the coordinator together identified a list of innovation-related teams. We conducted the first onsite survey with the help of the coordinators in a company meeting room during work hours, often before the team’s weekly (or monthly) meetings. For absent participants, we attained their contact information from their coordinators/team leaders and left both the survey and our self-addressed stamped envelopes. To increase response rates, we included both the endorsement letter from the CEO and a small gift, and distributed them together with our surveys. We conducted the second survey around 3 weeks later, to decrease the common-method bias (CMB). The first wave survey includes items on project commitment, learning from failure and control variables, and the second wave survey contains items on perceived shame after failure and personal control attribution for the last failed project.

The final sample consists of 140 teams (774 responses, including 634 from all team members and 140 team leaders) from 58 companies in the technology industry in mainland China, which participated in both surveys and had completed responses from all team members and team leaders. We excluded five companies which did not participate in the second survey or had missing responses from teams.

### Measures

As the definition of failure is not clear, previous studies generally defined it according to specific events. In this study, we defined project failure depending on the results of research projects. Following previous studies, we defined project failure as ‘the termination of an initiative to create organizational value that has fallen short of its goals’ (McGrath, 1999; Hoang and Rothaermel, 2005; Shepherd et al., 2011), and we gave this definition in the introduction section of our questionnaires.

#### Project Commitment

We measured project failure using Pinto et al.’s (1993) five-item scale with response options ranging from 1 (totally against) to 6 (totally agree), including ‘I feel fully responsible for achieving the project goals,’ ‘This project has the strong commitment of me,’ ‘I am proud of to be part of the project,’ ‘I am committed not only to my team, but to the overall project,’ ‘I value to be part of this project. The reliability estimate for the scale was 0.86.’

#### Learning from Failure

We measured learning from failure using an eight-item scale, including three items for personal dimensions and five items for project dimensions, developed by Shepherd et al. (2011), with response options ranging from 1 (totally against) to 6 (totally agree). Sample items include ‘I am more willing to help others deal with their failures,’ ‘I can more effectively run a project.’ The reliability estimate for the scale was 0.91.

#### Shame

We measured shame using Lickel et al.’s (2005) five-item scale with response options ranging from 1 (strongly disagree) to 6 (strongly agree), including ‘ashamed,’ ‘disgraced,’ ‘humiliated,’ ‘embarrassed,’ ‘shamefaced.’ Participants were asked to indicate the extent to which they agreed that these statements described their feelings after project failure. The reliability estimate for the scale was 0.93.

#### Personal Control

We measured personal control using Russell (1982) Causal Dimension Scale with response options ranging from 1 (totally against) to 6 (totally agree). A sample item is ‘The causes for the failure of the project are controlled by our team.’ Participants were asked to indicate the extent to which they agreed that these statements described attributions of project failure. The reliability estimate for the scale was 0.85.

#### Control Variables

Following previous studies (Mroczek and Kolarz, 1998; Hankin and Abramson, 2001), we included age, gender, resilience, education level, and team tenure as control variables. Resilience is the ability of a person to grow up in adversity, which is important for individuals to recover and maintain normal abilities to work and learn after stress so that people could continue to struggle for project goals (Campbell-Sills and Stein, 2007). Besides, as Shepherd and Cardon (2009) argue, resilience is positively related to personal emotional states after failure, which could work for the process of learning from failure. Therefore, we also employed resilience as the control variable which was measured by the 10-item scale validated by Campbell-Sills and Stein (2007). Besides, as education level is categorically variable, we created four dummy variables: d1 was coded as 1 = technical secondary, 0 = others; d2 was coded as 1 = junior college, 0 = others; d3 was coded as 1 = bachelor, 0 = others; d4 was coded as 1 = master, 0 = others. Gender was coded 1 for ‘male’ and 0 for ‘female.’

### Analytic Plan

First, we used AMOS 22.0 to do a confirmatory factor analysis (CFA) to examine the distinctiveness of our variables. Then, we conducted the single-level analysis with Mplus 7.4 and the multilevel regression with HLM 6.0. Results of single-level and multilevel analysis were reported in **Tables 3**, **4**, respectively. Specifically, when we conducted multilevel analysis, variables were grand-mean centered.

## Results

### Validity

**Table 1** shows the results of confirmatory factory analysis (CFA). As shown, our four-factor measurement model (project commitment, learning from failure, shame, and personal control) fits the data well (χ^2^ = 339.31, *df* = 84, CFI = 0.97, NFI = 96, RMSEA = 0.06). We also tested a null model, three-factor models, and a one-factor model. Our results show that the baseline model fits better than the other four alternative models, providing evidence of the construct distinctiveness of project commitment, learning from failure, shame, and personal control.

**Table 1:** Comparison of measurement model.

Model	Factors	χ^2^	df	NFI	CFI	RMSEA
Null model		8193.78	105			
Baseline model	Four factors	339.31	84	0.96	0.97	0.06
Model1	Three factors: project commitment and shame were combined into one factor	1679.44	87	0.80	0.80	0.16
Model2	Three factors: project commitment and personal control were combined into one factor	1509.73	87	0.82	0.82	0.15
Model3	Two: project commitment, shame and personal control were combined into one factor	2825.84	89	0.66	0.66	0.20


As we collected the predictor and outcome at the same time, we tested CFA with these two variables together as the one-factor model (χ^2^ = 943.01, *df* = 14, CFI = 0.76, NFI = 75, RMSEA = 0.29), and also test the two-factor model (χ^2^ = 66.28, *df* = 13, CFI = 0.98, NFI = 98, RMSEA = 0.07), indicating these two variables are distinguishable. Moreover, because of the limitation of cross-sectional data, we used Harman’s one-factor test (Podsakoff et al., 2003) to assess the presence of common method bias. Our results show that four factors explain a total of 74% variance, and no single factor accounts for the majority of the variance. Besides, the results of CFA also provide support. If the data had a serious common method bias problem, the single-factor could fit the data as well as the four-factor model (Huang, 2012). As mentioned above, the four-factor model fits better than other sample models, also indicating that common method bias is not serious in this study.

### Descriptive Statistics

**Table 2** shows the descriptive statistics (the mean, standard deviation, and reliability of variables) and the correlations among the variables. The data shows that project commitment is positively related to learning from failure (*r* = 0.48, *p* < 0.01), which preliminarily supports Hypothesis 1.

**Table 2:** Means, standard deviations, reliabilities, and correlations.

Variable	*M*	*SD*	1	2	3	4	5	6	7	8
(1) Gender	0.77	0.42								
(2) Age	3.44	0.17	-0.04							
(3) Years in the team	1.46	0.69	-0.04	0.52^∗∗^						
(4) Resilience	4.00	0.60	-0.06	0.03	-0.07	(0.88)				
(5) Project commitment	4.44	0.86	-0.02	0.01	-0.05	0.33^∗∗^	(0.86)			
(6) Learning from failure	4.58	0.84	-0.06	-0.01	-0.04	0.29^∗∗^	0.48^∗∗^	(0.91)		
(7) Shame	2.50	1.19	0.14^∗∗^	0.01	0.01	-0.06	-0.04	-0.09^∗∗^	(0.93)	
(8) Personal control	3.52	1.14	0.03	-0.12^∗∗^	-0.11^∗∗^	0.08^∗∗^	0.15^∗∗^	0.10^∗∗^	0.19^∗∗^	(0.85)


*^∗∗^*p* < 0.01.*

### Hypothesis Testing

Because our variables are nested within group, for instance, shame is a moral emotion strongly related to the social context (e.g., blame culture: Vince and Saleem, 2004; Khatri et al., 2009), we should estimate our models with a multilevel method. First, we tested the between-group and within-group variance in the outcome variable (i.e., learning from failure), and our results show that the intraclass correlation coefficient – ICC1 was 0.058, indicating that the single-level analysis is more appropriate. We reported the results of single-level regression in **Table 3**. To further examine whether the nested structure would affect our results, we also tested the multilevel model, and we reported multilevel regression analysis results in **Table 4**.

**Table 3:** Single-level estimates for models.

Variables	Model 1	Model 2	Model 3
Gender	-0.15*	-0.10	-0.10
Age	-0.02	-0.05	-0.05
d1	-0.20	-0.44	-0.40
d2	0.19	0.11	0.10
d3	0.06	0.01	0.02
d4	0.10	0.06	0.06
Years in the team	-0.02	-0.00	0.00
Resilience	0.41***	0.21***	0.19***
Project commitment		0.42***	0.43***
Shame		-0.05*	-0.06*
Person Control		0.03	0.02
Project Commitment × shame			0.05*
Project Commitment × personal control			0.05*
*R*^2^	0.09	0.25	0.26
Δ*R*^2^	0.09***	0.16***	0.01**


**^∗^p < 0.05; ^∗∗^p < 0.01; ^∗∗∗^p < 0.001; we created five dummy variables for education level: d1 was coded as 1 = technical secondary, 0 = others; d2 was coded as 1 = junior college, 0 = others; d3 was coded as 1 = bachelor, 0 = others; d4 was coded as 1 = master, 0 = others.**

**Table 4:** Multilevel estimates for models.

Variables	Null model	Model 4	Model 5	Model 6
Intercept	4.56***	3.07^∗∗∗^	3.73^∗∗∗^	3.80^∗∗∗^
**Level 1**				
Gender		-0.13 (0.06)	-0.07 (0.06)	-0.06 (0.05)
Age		-0.02 (0.23)	-0.01 (0.19)	-0.02 (0.21)
d1		-0.16 (0.68)	-0.30 (0.55)	-0.20 (0.98)
d2		0.17 (0.30)	0.07 (0.20)	0.06 (0.27)
d3		0.03 (0.29)	-0.02 (0.18)	-0.01 (0.25)
d4		0.07 (0.28)	0.04 (0.17)	0.04 (0.25)
Years in the team		-0.02 (0.05)	0.01 (0.05)	0.01 (0.04)
Resilience		0.41*** (0.06)	0.22*** (0.05)	0.21*** (0.06)
Project commitment			0.41*** (0.04)	0.41*** (0.04)
Shame			-0.05* (0.03)	-0.07* (0.03)
Person Control			0.03 (0.03)	0.02 (0.03)
Project Commitment × shame				0.08* (0.03)
Project Commitment × personal control				0.04 (0.04)
**Variance components**				
Within-team (Level 1) variance (ρ^2^)	0.662	0.613	0.432	0.428
Intercept (Level 2) variance (τ_00_)	0.04	0.04	0.01	0.02
*Pseudo R*^2^		0.14	0.30	0.01


*^∗^p < 0.05; ^∗∗∗^p < 0.001; we reported standard error (SE) in the bracket; we created five dummy variables for education level: d1 was coded as 1 = technical secondary, 0 = others; d2 was coded as 1 = junior college, 0 = others; d3 was coded as 1 = bachelor, 0 = others; d4 was coded as 1 = master, 0 = others; TPG = Team level project commitment; TS = Team level shame; TPC = Team level personal control.*

First, we conducted single-level analysis. Three hypotheses were tested by regression analysis for three steps: (1) putting in control variables (gender, age, education, years in the team, and resilience); (2) putting in project commitment, shame or personal control; (3) putting in two-way interaction terms of project commitment × shame or project commitment × personal control (the predictor and the moderator variables had been centered before they were entered into the interaction term). The results are presented in **Table 3**: (1) after controlling variables including gender, age, education level, years in the team, and resilience, project commitment is significantly and positively associated with learning from failure (*b* = 0.42, *p* < 0.001), which provides support to Hypothesis 1; (2) shame significantly moderates the influence of project commitment on learning from failure (*b* = 0.05, *p* < 0.05); (3) personal control significantly moderates the relationship between project commitment and learning from failure (*b* = 0.05, *p* < 0.05).

**FIGURE 2:**
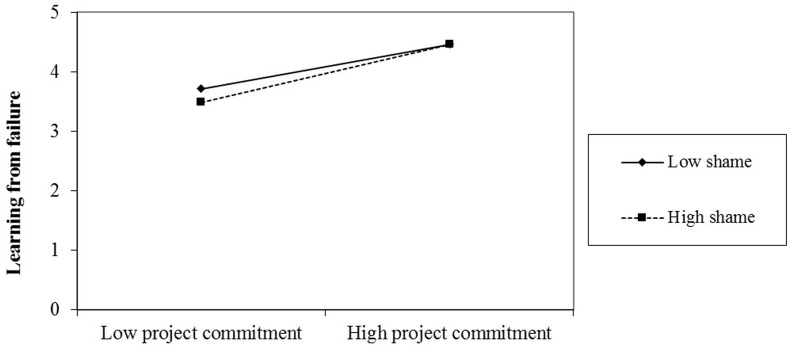
The moderating effect of shame.

**FIGURE 3:**
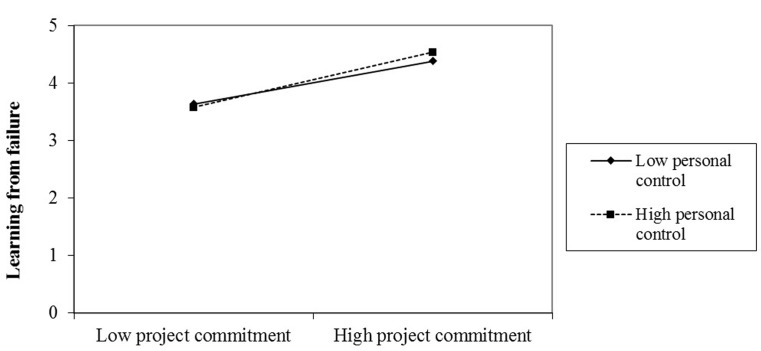
The moderating effect of personal control.

To further test Hypothesis 2 and Hypothesis 3, we considered a high level of shame as plus one standard deviation from its mean and, as to high level of personal control and regard and low sense of shame, as minus one standard deviation from the mean (**Figures 2**, **3**). As shown in these figures, the relationship between project commitment and learning from failure is more positive under the high personal control level (*b* = 0.48, *p* < 0.001) than that under the low personal control level (*b* = 0.38, *p* < 0.001); the relationship between project commitment and learning from failure is more positive under the high shame level (*b* = 0.48, *p* < 0.001) than that under the low shame level (*b* = 0.37, *p* < 0.001). Besides, the difference between the high and low level of personal control (*b* = 0.11, *p* = 0.031) as well as shame (*b* = 0.11, *p* = 0.028) is significant, providing further support for Hypothesis 2 and 3.

Second, we conducted multilevel regression. As shown in **Table 4**, the coefficients of project commitment (*b* = 0.41, *p* < 0.001; Model 5) and the two-way interaction term of project commitment and shame at level 1 (*b* = 0.08, *p* < 0.05; Model 6) are significant, providing support for Hypothesis 1 and 2. However, the coefficient of the two-way interaction term of individual project commitment and individual personal control is not significant. Thus, Hypothesis 3 was not supported.

Moreover, our results also showed that resilience is positively associated with learning from failure, providing empirical evidence for previous theoretical propositions, that resilience can help individuals take positive responses to cope with stressful events (Shepherd and Cardon, 2009; Waldman et al., 2011). To further confirm the role of resilience, we also tested three different models (resilience as the moderator; three-way interaction among project commitment, resilience, and shame; three-way interaction among project commitment, resilience, and personal control). However, coefficients of the two-way interaction term and three-way interaction terms are not significant.

## Discussion

The current study developed a model to explain why and when people learn from failed events, employing emotional bonds (i.e., project commitment) as the antecedent, cognition (i.e., personal control), and emotion (i.e., shame) as moderators. By analyzing the data, we came to these conclusions: (1) project commitment is positively related to learning from failure; (2) perceived shame after project failure moderates the aforementioned positive relationship in such a way that the relationship will be stronger when the level of shame is higher rather than lower; (3) perceived personal control positively moderates the relationship between project commitment and learning from failure in such a way that the relationship will be stronger when perceived personal control is higher rather than lower. However, this effect is not significant at between-group level.

### Theoretical Contribution

First, previous studies have proven that learning from failure is beneficial to team performance (Cannon and Edmondson, 2005) and innovation (Tahirsylaj, 2012), thereby accelerating the growth of a team and individuals. Therefore, how to stimulate people to learn from failure is becoming an important issue. However, there are few studies on the antecedents of learning from failure; thus, the present study aims to bridge this gap. As such, we successfully linked project commitment with learning from failure, and revealed the process by which employees with high levels of project commitment will learn more from failure because of their motivation to achieve project goals. Furthermore, our study first explores the relationship between emotional bonds and individuals’ behavioral response (i.e., learning from failure), enriching the literature on learning from failure by providing a new antecedent in a different angle.

Second, we emphasize the moderating roles of emotion and cognition during the learning process, and examine them together in one theoretical model. In the current model, attribution determines the direction and the resource allocation to face the failed event, while emotion acts as the catalyst during the process. Our model also expands the use of Zhao and Olivera’s (2006) framework of error reporting. After failure (error could be regarded as a minor failure), people have series of choices, such as reporting, learning, and withdrawing. However, before a behavioral response, people have experienced two phases involving cognition and emotion variables (Zhao and Olivera’s, 2006). In contrast to Zhao and Olivera’s study, which only focused on reporting, we applied this model to analyze another behavioral response (i.e., learning from failure), not only providing evidence for this model, but also giving a new path to analyze the behavior after a failure.

Moreover, the current study sheds new light on the positive role of shame in the learning process following failure. Past studies suggest that shame is the negative emotion associated with anger, rejection, and evasion (McGregor and Elliot, 2005), and it is the negative factor for learning from failure (Cohen et al., 2011). However, Bohns and Flynn (2013) propose that negative emotions may play a positive role in generating ‘learning from failure,’ while there is still no empirical study to examine the proposition. Our study examines the moderating effect of shame on the relationship between project commitment and learning from failure, and finds that it strengthens the aforementioned relationship, meaning that shame can play a positive role in Eastern cultures. This result not only enriches the original theory and provides empirical support for the previous theoretical proposition, but also provides new directions for future research and demonstrates an interesting aspect of emotions for researchers to explore under different cultures.

Moreover, our results also show that resilience is positively related to learning from failure, indicating the potential buffering role of resilience. We suppose that resilience may exert a positive effect on an individual’s cognition and emotions after failure, such that people with high resilience may have higher self-efficacy or confidence, thereby processing information more efficiently and learning more from past failures. However, Sitkin (1996) also proposed that resilience could be a long-term positive outcome of failed events rather than an antecedent or moderator. Researchers should examine the relationship between resilience and learning from failure further.

Finally, our study provides a new model to explain an individual’s behavioral response after failure. Following Zhao and Olivera (2006), we regard learning from failure as one certain behavioral response after failed events. According to previous studies, the emotional bond is closely related to individual behavior, and employees with a high commitment toward projects or teams are more likely to take measures to improve the situation and avoid failing again. Most commonly, therefore, emotional bonds lead to desirable outcomes. However, it also contributes to negative outcomes. For example, employees may blame another’s mistakes because of their own commitment to projects, thereby decreasing the whole team’s learning behavior (Tjosvold et al., 2004). Thus, our model takes boundary variables into account. We find that cognition in the detection and assessment phase has an impact on individual behavioral responses. For instance, if an individual believes the causes of failure are under their control, they will take measures focusing on themselves. Besides, emotion will positively or negatively influence individual motivations to improve.

Taken together, this framework (see **Figure 1**) could be expanded and applied in more contexts. For example, group identity, and supervisor-subordinate relationships as emotional bonds could increase employee motivations to improve (i.e., the total resources to make an effort). Individuals with different cognitions will make their own resources dividing plans, and determining their behavioral response types. Besides, other emotions could also be included in this framework, such as fear, guilt, and embarrassment (Zhao and Olivera, 2006).

### Practical Contributions

Our study makes contributions to managerial practices as follows: First, our study finds the positive relationship between project commitment and learning from failure, which suggests teams and managers should provide various forms of support for employees (e.g., employee-assistance programs, team activities) to enhance their sense of belonging.

Besides, the present study finds that shame can be a positive element in some cases. Thus, team leaders should not always restrain negative emotions after failure. In contrast, they are supposed to guide team members to manage negative emotions more effectively by emotional management. What is more, for leaders, managers could foster the cultural values of organizations to create a friendly environment for learning from failure. However, it is noteworthy that inappropriate measures may lead to ethical issues in business. Too many negative emotions may bring harm to employees (Bohns and Flynn, 2013), so managers should be careful to intervene in staff emotions.

Finally, attribution is an important factor in the process of learning from failure. The results of our study show that personal control can strengthen a project’s commitment impact on learning from failure. For this reason, managers should help employees form a correct attributional pattern and improve their sense of personal control. Managers could also increase employee autonomy through delegation and provide effective training to improve employee ability, which is beneficial for their sense of personal control.

### Limitations and Future Research

In addition to the above contributions, our study has some limitations. First, the data we used were only collected from R&D teams in China, and the scales we used came from international maturity scales which may not fully match Chinese ones. Previous studies suggest that there are broad differences between Eastern and Western cultures about shame, especially between Confucian and American culture, such as, proneness to experience guilt or shame (Bedford and Hwang, 2003). Researchers found that Westerners are more likely to be driven by guilt. In contrast, Easterners emphasize shame, and they take measures to overcome it (Bedford and Hwang, 2003). Thus, future researchers could investigate the generalisability of our results in different areas and teams with indigenous scales and try to conduct cross-cultural research. What is more, shame in the current study acts as the state-like variable, describing a respondent’s emotions after failure. However, shame could also be a trait characteristic. Therefore, it is necessary to pay attention to the trait of shame in the future, for instance, taking it as the control variable when we explore emotion and behavior after failed events.

Second, we collected the predictor and outcome at the first survey, and collected moderators in the second survey. Although emotion after failure is closely related to project commitment and learning, meaning that it is necessary to measure these variables at different time points, collecting the predictor and outcome at the same time may lead to CMB. Researchers can make a three-wave investigation, separating the measurement of predictor, moderators, and outcome, or make longitudinal exploration in the future to decrease the CMB. Besides, scholars also can ask employee supervisors or co-workers to assess their learning behavior, and can use physical indexes to measure emotions, such as heart rate (Matta et al., 2017), to minimize the CMB (Podsakoff et al., 2003).

Third, results of multilevel analysis show that the moderating role of personal control (Hypothesis 3) is not significant, while it is supported at the within-group level, indicating the nested structure influences moderating the role of personal control. The difference between within-group and between-group analysis provides a new direction for future studies, and scholars could examine this phenomenon with stricter research designs, combining laboratory experiments and field study in the future.

Finally, we only examined the moderating effect of shame and personal control on the project commitment-learning from failure relationship. Future studies could examine more variables based on our model, such as other emotions (e.g., embarrassment: Bohns and Flynn, 2013), other dimensions of attribution theory (e.g., the locus dimension: Standing et al., 2006), and contextual variables (Tolli and Schmidt, 2008). Specifically, we should put more effort in examining the role of emotion after failure (Baumeister et al., 2007). Shepherd and Cardon (2009) state that emotional reactions mediate the relationship between project failure and behavioral responses. To further explore the role of emotion after failure, employees can complete diaries tracing emotion changes over a period (such as a day, week, or a month: Quinn et al., 2012). Besides, we did not consider mediating variables in our study. Future researchers may discuss the mediating variable on this relationship, for instance, psychological safety (Carmeli and Gittell, 2009), thereby illuminating the process.

## Conclusion

Given the important role of learning from failure in the commercial world, previous studies have explored the outcomes of learning from failure from different angles. The current study examined the relationship between project commitment and learning from failure. By doing this, we contributed to the further development of learning from failure. At the same time, the present study also investigates shame and personal control as moderators of project commitment-learning from failure relationship, which provides a new direction for the future.

## Ethics Statement

All procedures performed in studies involving human participants were in accordance with the ethical standards of the institutional and/or national research committee and with the 1964 Helsinki declaration and its later amendments or comparable ethical standards with written informed consent from all subjects. This study was carried out in accordance with the Declaration of Helsinki and ethical guidelines and approved by the Human Research Ethics Committee (HREC) at the Beijing Normal University.

## Author Contributions

WW and BW substantially contributed to the conception and the design of the work as well as in the analysis and interpretation of the data. As KY and CY prepared the draft. WY and SS reviewed it critically and gave important intellectual input.

## Conflict of Interest Statement

The authors declare that the research was conducted in the absence of any commercial or financial relationships that could be construed as a potential conflict of interest. The reviewer DU and handling Editor declared their shared affiliation.
